# Reduction of Thrombus Burden With Short‐Term, Low‐Dose Rivaroxaban Use in Acute Myocardial Infarction: The ARISE‐ARMYDA 7 Randomized Trial

**DOI:** 10.1161/JAHA.125.041993

**Published:** 2025-11-06

**Authors:** Marco G. Mennuni, Leonardo Grisafi, Gianluca Anastasia, Martina Solli, Vincenzo Galiffa, Matilde Villa, Manuel Bosco, Enrico Incaminato, Roberta Rosso, Francesco De Crescenzo, Domenico D’Amario, Rocco Vergallo, Rebecca Rippa, Giuseppe Patti

**Affiliations:** ^1^ Department of Translational Medicine University of Eastern Piedmont Novara Italy; ^2^ Maggiore della Carità Hospital Novara Italy; ^3^ Department of Internal Medicine and Medical Specialties University of Genoa Genoa Italy; ^4^ IRCCS San Martino Polyclinic Hospital Genoa Italy

**Keywords:** optical coherence tomography, percutaneous coronary intervention, rivaroxaban, STEMI, thrombus burden, Coronary Circulation, Percutaneous Coronary Intervention, Pharmacology, Thrombosis

## Abstract

**Background:**

Managing large coronary thrombus burden (LCTB) in patients with ST‐segment–elevation myocardial infarction (STEMI) remains challenging. Although deferred stenting emerged to potentially improve outcomes in this high‐risk population, the optimal antithrombotic regimen remains unclear. The ARISE‐ARMYDA 7 (Alternative Anti‐Thrombotic Pathways in Acute Myocardial Infarction–Antiplatelet Therapy for Reduction of Myocardial Damage 7) trial evaluated low‐dose rivaroxaban in addition to dual antiplatelet therapy for LCTB reduction in patients with STEMI managed with deferred stenting.

**Methods:**

This single‐center, randomized, pilot study included patients with STEMI with angiographic evidence of LCTB undergoing primary percutaneous coronary intervention and deferred stenting. Patients were randomized to rivaroxaban 2.5 mg BID plus aspirin and ticagrelor or aspirin plus ticagrelor alone. Thrombus burden was assessed by optical coherence tomography at baseline and after 5 to 7 days of treatment. The primary end point was reduction of thrombus score after this period.

**Results:**

A total of 40 patients with STEMI and LCTB were randomized 1:1. Posttreatment thrombus score at re‐optical coherence tomography imaging was significantly lower in the rivaroxaban arm (39 [27–52] versus 82 [50–111] in controls, *P*=0.005). Relative reduction of the thrombus score versus baseline was greater with rivaroxaban use (61% [50%–81%] versus 36% [0%–50%], *P*=0.002). The relative thrombus volume decrease was 77% with rivaroxaban versus 39% in the control arm (*P*=0.001). Deferred stenting was safe, with no abrupt vessel closures, distal embolization, or no reflow. Clinical outcomes at 30 days, including major adverse cardiovascular events and bleeding complications, were not significantly different.

**Conclusions:**

The ARISE‐ARMYDA 7 trial shows that adding low‐dose rivaroxaban to dual antiplatelet therapy significantly reduces thrombus burden in patients with STEMI with LCTB, while maintaining a favorable safety profile.

**Registration:**

URL: https://www.clinicaltrialsregister.eu/; Unique identifier: EudraCT 2020–005156‐38.

**Nonstandard Abbreviations and Acronyms**
ARISE‐ARMYDA 7: Alternative Anti‐Thrombotic Pathways in Acute Myocardial Infarction–Antiplatelet Therapy for Reduction of Myocardial Damage 7
ATLAS ACS 2‐TIMI 51: Anti‐Xa Therapy to Lower Cardiovascular Events in Addition to Standard Therapy in Subjects With Acute Coronary Syndrome 2– Thrombolysis In Myocardial Infarction 51
BARC: Bleeding Academic Research Consortium
COMPASS: Cardiovascular Outcomes for People Using Anticoagulation Strategies
DAPT: dual antiplatelet therapy
LCTB: large coronary thrombus burden
TAPAS: Thrombus Aspiration During Percutaneous Coronary Intervention in Acute Myocardial Infarction
TASTE MI: Thrombus Aspiration in ST‐Elevation Myocardial Infarction
TIMI: Thrombolysis In Myocardial Infarction
TOTAL: Trial of Routine Aspiration Thrombectomy With PCI Versus PCI Alone in Patients With STEMI

**Clinical Perspective**

**What Is New?**

- The ARISE‐ARMYDA 7 (Alternative Anti‐Thrombotic Pathways in Acute Myocardial Infarction–Antiplatelet Therapy for Reduction of Myocardial Damage 7) trial is the first randomized study to demonstrate that the early addition of low‐dose rivaroxaban (2.5 mg BID) to standard dual antiplatelet therapy significantly reduces thrombus burden in patients with ST‐segment–elevation myocardial infarction and large coronary thrombus burden undergoing primary percutaneous coronary intervention with deferred stenting.
- Using high‐resolution optical coherence tomography, we showed a greater reduction in thrombus volume, area, and length with rivaroxaban compared with dual antiplatelet therapy alone, without increasing major bleeding risks.

**What Are the Clinical Implications?**

- Early addition of low‐dose rivaroxaban to dual antiplatelet therapy in patients with ST‐segment–elevation myocardial infarction with large coronary thrombus burden safely enhances thrombus resolution before deferred stenting, potentially reducing embolization‐related microvascular injury and improving outcomes.

Thrombus formation on the surface of atherosclerotic plaques is a key moment in the pathogenesis of an acute myocardial infarction (AMI), driven by platelet and coagulation pathways activation.^1^ Primary percutaneous coronary intervention (PCI) with stent implantation, combined with epicardial coronary reperfusion and dual antiplatelet therapy (DAPT), represent the standard treatment in patients with ST‐segment–elevation myocardial infarction (STEMI).^2^, ^3^ The management of STEMI prioritizes a prompt diagnosis, early administration of antiplatelet drugs, and immediate mechanical retrieval of the vessel patency by stent deployment.^2^, ^3^ However, the presence of a large coronary thrombus burden (LCTB) in patients with STEMI poses significant challenges, because it can compromise primary PCI results because of distal embolization and microvascular obstruction, leading to larger infarcts and worse clinical outcomes.^4^, ^5^

Thus, the deferral of PCI following a short period of antithrombotic therapy aimed at decreasing the thrombus burden has been proposed in selected cases to improve prognosis.^6^ In particular, a strategy alternative to the standard approach may be represented by minimally invasive procedures to restore a coronary flow sufficient for myocardial metabolism, followed by pharmacological neoadjuvant therapy to reduce thrombus burden and deferred stenting. Although this approach has shown promising results in reducing coronary flow impairment and improving left ventricular function, it has not demonstrated a clinical or survival benefit when aspirin, clopidogrel, heparin, and anti‐IIb/IIIa agents were investigated to decrease thrombus burden.^7^ Thus, the optimal antithrombotic approach to prepare the culprit coronary lesion before deferred PCI in patients with STEMI remains unclear.

Thrombus formation following plaque rupture/erosion involves complex interactions between platelet activation and fibrin generation.^8^ Thrombin plays a key role in this process by activating platelets and converting fibrinogen to fibrin, leading to thrombus formation.^9^ This interplay may support a therapeutic approach of dual‐pathway inhibition targeting both coagulation and platelet activity. In the warfarin era, combining anticoagulation with antiplatelet therapy in patients with acute coronary syndrome (ACS) reduced the occurrence of thrombotic events, but was challenging for routine use due to a high bleeding risk and difficult management.^10^ The introduction of direct oral anticoagulants with improved safety profiles and ease of use has advanced newer therapeutic options for thrombus management also in the setting of ACS. In particular, the ATLAS ACS 2‐TIMI 51 (Anti‐Xa Therapy to Lower Cardiovascular Events in Addition to Standard Therapy in Subjects With Acute Coronary Syndrome 2–Thrombolysis In Myocardial Infarction 51) trial compared low‐dose rivaroxaban, a direct factor Xa inhibitor providing the aforementioned dual‐pathway inhibition, versus placebo, with the treatment given on top of standard DAPT among patients with recent ACS. The use of rivaroxaban here reduced the incidence of cardiovascular events during follow‐up, but increased the occurrence of bleeding complications.^11^

To date, no study has addressed the issue of whether early initiation of an oral anticoagulant treatment is able to reduce thrombus burden over the short term in patients with acute STEMI. In the ARISE‐ARMYDA 7 (Alternative Anti‐Thrombotic Pathways in Acute Myocardial Infarction‐Antiplatelet Therapy for Reduction of Myocardial Damage 7) trial we hypothesized that a differential antithrombotic strategy targeting both platelets and coagulation pathways may effectively and safely decrease thrombus burden in patients with STEMI with LCTB. In particular, the aim was to investigate whether adding rivaroxaban 2.5 mg twice daily to DAPT (aspirin plus ticagrelor) in patients with STEMI after primary PCI without stenting provides an additional reduction of thrombus burden at the site of the culprit lesion as preparation to deferred coronary stenting.

## METHODS

The ARISE‐ARMYDA 7 trial is a randomized, open‐label, controlled pilot study performed on patients with STEMI and an LCTB admitted at Maggiore della Carità Hospital in Novara, Italy. The study adheres to the Consolidated Standards Of Reporting Trials guidelines for pilot and feasibility trials.

The data that support the findings of this study are available from the corresponding author upon reasonable request.

Patients presenting with STEMI <24 hours from symptom onset, undergoing primary PCI, and demonstrating LCTB at coronary angiography were eligible for inclusion. LCTB was defined as a thrombus grade 4 (definite thrombus with the largest dimension being ≥2 vessel diameters) or grade 5 (total thrombotic occlusion, as determined by angiographic criteria).^12^ Other key inclusion criteria were a culprit vessel reference diameter ≥3.0 mm at angiography and a successful coronary revascularization with primary PCI, defined as the simultaneous presence of the following 3 criteria: restoration of TIMI (Thrombolysis In Myocardial Infarction) flow grade ≥2, residual stenosis <50% at angiography, and ≥50% ST‐segment–elevation resolution at electrocardiogram after primary PCI.

Exclusion criteria encompassed:

1. High bleeding risk, defined as presence of clinically significant active bleeding, conditions with high risk of major bleeding, such as recent or ongoing gastric ulceration, presence of cancer with high risk of bleeding, recent brain or spinal trauma/surgery/hemorrhage, known or suspected esophageal varices, arteriovenous malformations, vascular aneurysms or major intraspinal or intracerebral vascular dysfunctions, and liver disease with coagulopathy and Child‐Pugh B/C class
2. Use of femoral artery approach for coronary angiography and intervention
3. Contraindication to oral anticoagulation
4. Severe renal impairment, defined as an estimated glomerular filtration rate <15 mL/min per 1.73 m^2^
5. Cardiogenic shock
6. Known hypersensitivity to the study drug
7. Recent stroke (<6 months)
8. Pregnancy

**Figure 1:**
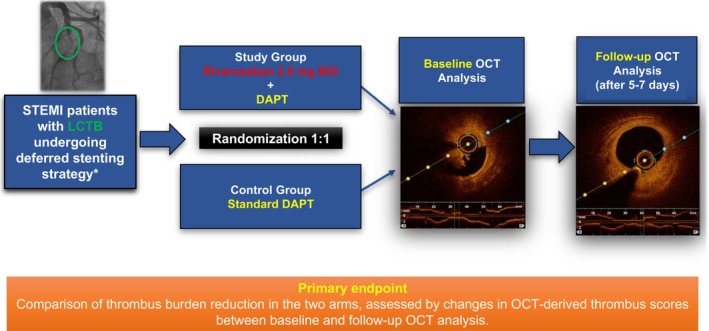
Study design. *Patients meeting the following inclusion criteria were enrolled: (1) culprit vessel reference diameter ≥3.0 mm at angiography; (2) successful coronary revascularization with primary percutaneous coronary intervention, defined by postprocedural Thrombolysis In Myocardial Infarction flow ≥2, angiographic residual stenosis <50%, and ≥50% ST‐segment resolution at electrocardiography. DAPT indicates dual antiplatelet therapy; LCTB, large coronary thrombus burden; OCT, optical coherence tomography; and STEMI, ST‐segment–elevation myocardial infarction.

Patients with STEMI with LCTB in the culprit lesion at baseline coronary angiography performed for primary PCI meeting the enrolment criteria were randomized (1:1) to the intervention arm (rivaroxaban 2.5 mg BID on top of DAPT with aspirin and ticagrelor) or control arm (DAPT alone with aspirin and ticagrelor) (Figure 1). Randomization was implemented via a secure web‐based system to ensure allocation concealment. An independent investigator generated the random allocation sequence using a computer program. Sequentially numbered, opaque, sealed envelopes were used to maintain blinding during allocation. In both groups, ticagrelor was given as a 180 mg loading dose followed by 90 mg BID, and aspirin as a 500 mg intravenous load (in patients not on chronic aspirin), followed by oral 100 mg daily. Both these antiplatelet agents were administered in the catheterization laboratory before primary PCI. In all patients, such a procedure included thrombus aspiration and balloon angioplasty to restore the flow to TIMI grade ≥2, but without stent implantation. A baseline optical coherence tomography (OCT) imaging analysis, specifically focused to thrombus measures at the site of the culprit lesion, was performed after coronary vessel recanalization. The use of glycoprotein IIb/IIIa inhibitors was left to the operator’s discretion. In the intervention arm, rivaroxaban was initiated in the intensive care unit immediately after primary PCI. All patients received 5 to 7 days of treatment with DAPT plus rivaroxaban (intervention arm) or DAPT alone (control arm). The use of heparin was allowed during the baseline and control procedures, whereas its use was not permitted during the intercurrent treatment period. After 5 to 7 days, angiography and OCT imaging analysis were repeated to reassess thrombus burden at the site of the culprit lesion and guide the deferred PCI, which was immediately completed in all patients with or without stent implantation. Repeat OCT imaging provided detailed quantitative assessments of posttreatment thrombus measurements, including size, area, volume and length. After the deferred re‐PCI, DAPT with aspirin and ticagrelor was continued, regardless of the randomization assignment for the study. A clinical follow‐up was performed at 30 days. Key assessments included demographic, clinical, echocardiographic and laboratory data, alongside OCT and angiographic findings in all participants.

### OCT Image Acquisition and Analysis

**Figure 2:**
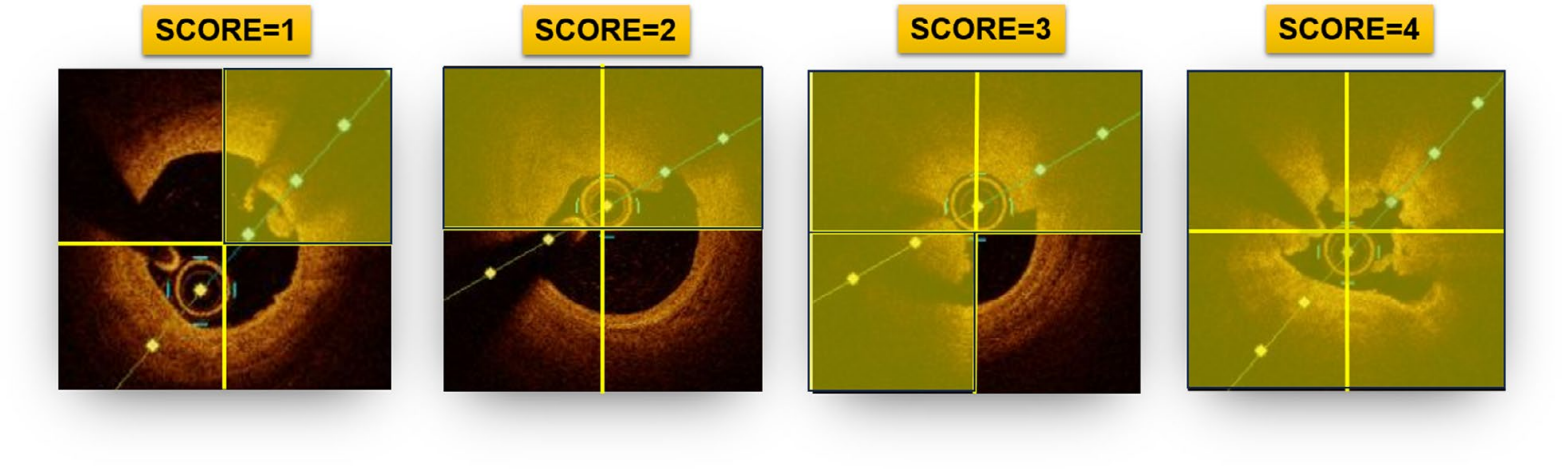
Representation of thrombus score. The thrombus score is determined by counting the number of quadrants occupied by thrombus in each cross‐section during a pullback. Using this approach, in every cross‐sectional optical coherence tomography image, thrombus at the culprit lesion is categorized as absent or involving 1, 2, 3, or 4 quadrants. In the example, the total thrombus score, calculated as the sum of quadrant involvement across all cross‐sections, is *x*.

OCT pullback of the culprit vessel was performed after intracoronary administration of nitroglycerin (100–200 μg), using an Fourier Domain‐OCT system (ILUMIEN OPTIS, Abbott Vascular). An automated pullback was performed at a speed of 36 mm/s (180 frames/s) using a Dragonfly OptisTM imaging catheter (Abbott Vascular). Analysis was performed at 0.2 mm intervals by 2 independent investigators (G.A., Re.Ri.) blinded to the randomization arm and angiographic, clinical, and laboratory data, using proprietary software for offline analysis (Aptivue Offline Review Workstation, Abbott Vascular). In case of discordance, a consensus was achieved with a third investigator (R.V.). Thrombus was defined as an irregular mass protruding into the lumen attached to the vessel wall or discontinuous from its surface, and categorized as: (1) red thrombus, defined as a highly backscattering mass with high attenuation; (2) white thrombus, appearing as a less backscattering, homogeneous mass with low attenuation; or (3) mixed thrombus. In each frame, the number of quadrants encroached by thrombus was determined. Each frame was additionally analyzed for the assessment of thrombus area, calculated as lumen area–flow area. Flow area represented the residual coronary lumen not occupied by thrombus. Thrombus area values were then averaged along the entire longitudinal segment of the culprit lesion in each patient. Thrombus length was measured as the distance between the first and last frames containing thrombus.^13^ Based on the above‐mentioned measurements, the following indices of thrombus burden were derived: thrombus score defined as the sum of the number of quadrants containing thrombus in each analyzed cross‐section along the lesion length (Figure 2)^14^ and thrombus volume (mm^3^) defined as the mean thrombus area (mm^2^)×thrombus length (mm).^13^

### End Points

The primary end point was the comparison of thrombus burden reduction in the 2 arms, assessed through changes in OCT‐derived thrombus scores at repeat imaging (after 5–7 days) compared with baseline imaging.

Secondary End Points

The following secondary end points were evaluated in the 2 arms:

1. Reduction of thrombus area, thrombus length, and thrombus volume, as assessed through OCT‐derived changes of the respective parameters at repeat imaging compared with baseline
2. Descriptive distribution of thrombus types (baseline and posttreatment)
3. Clinical outcome for incidence of major adverse cardiovascular events (a composite of cardiovascular death, myocardial infarction, stroke, or unplanned new target vessel revascularization), evaluated at the 30‐day follow‐up, alongside for occurrence of bleeding complications, classified by the BARC (Bleeding Academic Research Consortium) scale.^15^

The study adhered to the ethical principles outlined in the Declaration of Helsinki. Approval was obtained from the ethics committee of Maggiore della Carità Hospital in Novara (CE 269/20) and the Italian Medicines Agency (authorization number 27886 08/03/2021). The trial was registered in the European Union Clinical Trials Register (number 2020‐005156‐38). All participants provided written informed consent before enrollment.

### Statistical Analysis

We hypothesized that the addition of rivaroxaban to DAPT may result in a greater reduction of thrombus burden compared with standard DAPT alone. Sample size calculation was based on the primary end point of thrombus score decrease by OCT imaging, analyzed by a paired pre‐ and posttreatment design. In a previous study on patients with AMI treated with antithrombotic therapy, but without coronary stenting, patients received 3 days of heparin plus DAPT, followed by DAPT alone (aspirin plus ticagrelor) up to 30 days;^16^ here, the pretreatment thrombus score was 66±49 units, with a posttreatment score of 18 units.^16^ Given the shorter treatment duration in our study, we expected a higher posttreatment thrombus score (40 units) in the control group. We hypothesized that adding rivaroxaban to DAPT would lead to a greater thrombus burden reduction compared with DAPT alone. Specifically, we assumed a baseline mean thrombus score of 66 units (with an SD of ±49) in both study arms. In the control group, the expected posttreatment score was 40 units, corresponding to a relative reduction of 39%. In the rivaroxaban group, we hypothesized a more pronounced reduction of the posttreatment score (from 66 to 11 units, reflecting an 83% relative decrease). This anticipated difference between the 2 groups, based on an approximately 2‐fold greater relative thrombus reduction in the rivaroxaban group, was the basis for the sample size. To achieve 80% power with a 5% significance level for a 2‐sided paired *t* test, the required sample size was 19 patients per group (38 total). To account for a 15% dropout rate or unusable OCT data, the final sample size was adjusted to 44 patients, ensuring sufficient power to detect a meaningful thrombus reduction and establish the potential benefit of rivaroxaban addition.

Continuous variables were expressed as mean±SD if normally distributed, and comparisons between groups were performed using the Student *t* test. The Levene test was used to assess homogeneity of variances before Student *t* test application. The normality of the data was assessed by the Shapiro‐Wilk test. If the data were not normally distributed, continuous variables were expressed as median and interquartile range (IQR), and comparisons between groups were done using the Mann‐Whitney *U* test. In addition to standard nonparametric testing, permutation tests using 10 000 Monte Carlo replications, were conducted to derive more robust and assumption‐free inference. Categorical variables are presented as absolute numbers (percentages) and compared by the Fisher exact test, if the expected frequency was <5; otherwise, the χ^2^ test was used. Effect sizes were also reported by Cliff’s ∆ where appropriate to support interpretation of clinical impact. Patient data were collected in pseudonymized form using electronic case report forms and stored securely.

Statistical analyses were conducted using Stata version 18.0 (StataCorp, College Station, TX).

## RESULTS

**Figure 3:**
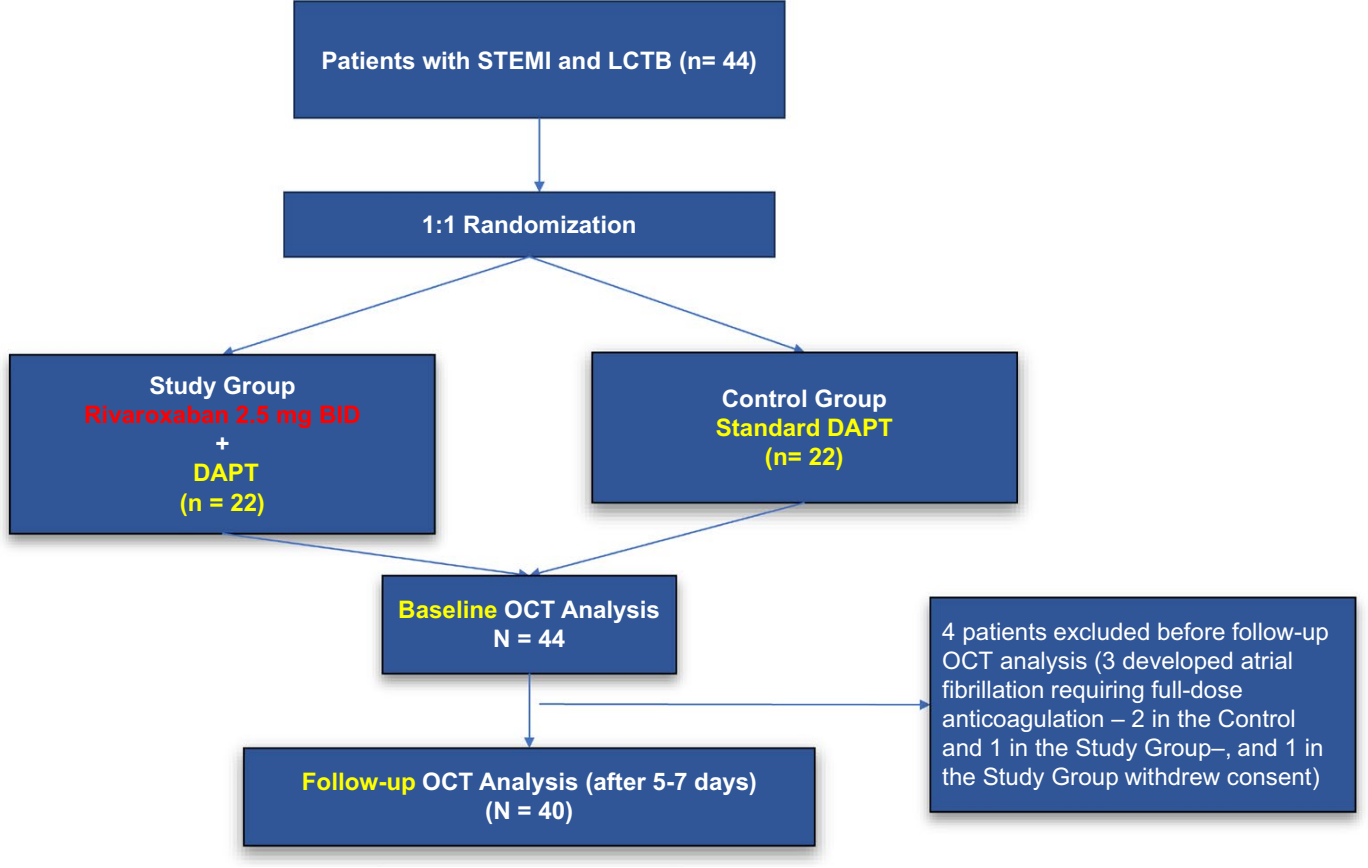
Study flowchart. DAPT indicates dual antiplatelet therapy; LCTB, large coronary thrombus burden; OCT, optical coherence tomography; and STEMI, ST‐segment–elevation myocardial infarction.

From November 23, 2021 to November 7, 2024, a total of 56 patients were initially screened for eligibility. Out of those patients, 12 patients (21%) were excluded before randomization due to failure to achieve angiographic residual stenosis <50% following primary PCI with balloon angioplasty and/or thrombectomy plus restoration of TIMI flow grade ≥2 and ≥50% resolution of ST‐segment elevation at electrocardiogram. Out of these 44 randomized patients, a total of 4 patients were then excluded after the randomization before the repeat OCT analysis, because 3 developed new‐onset atrial fibrillation requiring full‐dose anticoagulation (n=2 in the control arm, n=1 in the rivaroxaban arm) and 1 withdrew the consent. Thus, a total of 40 patients, receiving low‐dose rivaroxaban‐based therapy on top of aspirin plus ticagrelor (n=20, intervention arm) or DAPT with aspirin plus ticagrelor alone (n=20, control arm), were included as the final study population. All interventions were performed under standard anticoagulation protocols, including unfractionated heparin during both the index and deferred procedures, and a radial artery approach was used in all cases. Figure 3 shows the flowchart of participant recruitment, randomization, and retention. The follow‐up period ended on December 7, 2024.

The majority of patients in both groups had an anterior infarction, with left anterior descending artery being the culprit vessel and preserved left ventricular function (Table 1). In the overall population, the mean age was 62.3 years, the prevalence of female sex was 12%, the median time from symptom onset to catheterization laboratory was 180 minutes, the frequency of multivessel disease was 58%, and the median angiographic thrombus score by the TIMI thrombus scale was 5 out of 5. Angiographic TIMI flow grade 0 to 1, indicative of severely impaired perfusion, was observed in 35 patients (88%). As per protocol, no stent implantation was performed during primary PCI, which consisted of balloon angioplasty in 32 cases (80%) and/or thrombus aspiration in 26 cases (65%) (Table 1). The percentage distribution of grade 4 thrombus was overall 33% (13 patients) and of grade 5 thrombus was 66% (27 patients). In the overall population of 40 patients, plaque rupture was overall identified in 60% of cases, whereas plaque erosion was present in 40%. Post‐primary PCI outcomes demonstrated a high degree of procedural success, with a median TIMI flow grade of 3 (IQR, 3–3), and a residual mean TIMI thrombus score of 3.0±0.7. Distal embolization occurred in 2 patients (5%), and a transient no‐reflow was observed in 2 cases (5%).

**Table 1:** Demographic, Clinical, and Procedural Data

	Overall, N=40	Rivaroxaban arm, n=20	Control arm, n=20	Statistic*	*P* value
Demographic features					
Age (y)	62.3±11.9	63.6±12.3	61.0±11.2	*t*=0.69	0.50
Female sex	5 (12)	2 (10)	3 (15)	χ^2^=0.23	0.63
BMI (kg/m^2^)	26.3 [24.1–27.7]	26.6 [22.5–28.4]	26.2 [24.5–27.5]	*U*=388	0.56
Clinical data					
Systemic hypertension	20 (50)	12 (60)	8 (40)	χ^2^=1.60	0.20
Diabetes	4 (10)	1 (5)	4 (20)	χ^2^=2.06	0.15
Previous MI	6 (15)	3 (15)	3 (15)	χ^2^=0	1.00
Peripheral arterial disease	4 (10)	3 (15)	1 (5)	χ^2^=1.11	0.29
Chronic kidney disease	3 (8)	2 (10)	1 (5)	χ^2^=0.36	0.54
Anterior MI	19 (48)	7 (35)	12 (60)	χ^2^=2.51	0.11
Killip class at admission	1 [1–1]	1 [1–1]	1 [1–1]	χ^2^=5.26	0.18
Echocardiographic LVEF <40% at admission	5 (13)	2 (10)	3 (15)	χ^2^=0.23	0.63
Time from symptom onset to catheterization laboratory (min)	180 [115–480]	180 [115–480]	180 [90–480]	*U*=401	0.80
Angiography data at baseline (during primary PCI)					
Multivessel disease	23 (58)	12 (60)	11 (55)	χ^2^=0.10	0.74
Culprit vessel				χ^2^ =2.20	0.73
LAD	18 (45)	9 (45)	9 (45)		
LCA	10 (25)	4 (20)	6 (30)		
RCA	12 (30)	7 (35)	5 (25)		
TIMI flow score	0 [0–0]	0 [0–1]	0 [0–0]	*U*=421	0.68
TIMI thrombus score	5 [5–5]	5 [4–5]	5 [5–5]	*U*=446	0.77
Primary PCI data					
Thrombus aspiration	26 (65)	13 (65)	13 (65)	χ^2^=0	1.00
Balloon angioplasty	32 (80)	16 (80)	16 (80)	χ^2^=0	1.00
Distal embolization	2 (5)	1 (5)	1 (5)	χ^2^=0	0.97
No‐reflow	2 (5)	2 (10)	0 (0)	χ^2^=2.22	0.13
Postprocedural TIMI flow	3 [3–3]	3 [3–3]	3 [3–3]	*U*=430	0.38
Postprocedural reference vessel diameter (mm)	3.39±0.64	3.40±0.53	3.38±0.78	*t*=0.09	0.93
Postprocedural diameter stenosis (%)	57.1±15.0	59.3±9.5	54.5±19.9	*t*=0.77	0.45
Postprocedural lesion length (mm)	17.20 [10.74–23.90]	16.09 [11.03–20.70]	21.76 [10.10–42.68]	*U*=150	0.47
Periprocedural use of IIb‐IIIa inhibitors	30 (75)	13 (65)	17 (85)	χ^2^=2.13	0.14
Concomitant therapy after primary PCI					
Aspirin	40 (100)	40 (100)	40 (100)	…	1
Ticagrelor	40 (100)	40 (100)	40 (100)	…	1
ACE inhibitors/sartans	37 (93)	18 (90)	19 (95)	χ^2^=0.36	0.55
β‐Blockers	37 (93)	18 (90)	19 (95)	χ^2^=0.62	0.55
Statins	40 (100)	40 (100)	40 (100)	…	1
PCSK9i	9 (23)	5 (25)	4 (20)	χ^2^=0.14	0.70
Posttreatment reangiography data (at the time of deferred PCI)					
TIMI flow score	3 [3–3]	3 [3–3]	3 [3–3]	*U*=390	0.15
Diameter stenosis (%)	49.0 [44.5–58.0]	53.0 [47.0–58.0]	47.0 [31.0–55.0]	*U*=186	0.17
Lesion length (mm)	13.92±8.02	13.97±6.17	13.86±10.10	*t*=0.03	0.97

Data are expressed as absolute numbers (percentages) for categorical variables and mean±SD for continuous variables with normal distribution or median [interquartile range] for continuous variables with nonnormal distribution.

ACE indicates angiotensin‐converting enzyme; BMI, body mass index; LAD, left anterior descending; LCA, left circumflex artery; LVEF, left ventricular ejection fraction; MI, myocardial infarction; PCI, percutaneous coronary intervention; PCSK9i, proprotein convertase subtilisin/kexin type 9 inhibitors; RCA, right coronary artery; and TIMI, Thrombolysis In Myocardial Infarction.

* The Statistic column reports the value and type of statistical test used for between‐group comparisons: *t*=Student *t* test statistic and *U*=Mann‐Whitney *U* test statistic.

The 2 arms at baseline were similar in terms of age, sex, cardiovascular risk factors, comorbidities, clinical status, time from symptom onset to catheterization laboratory, left ventricular function, type and extension of coronary artery disease, baseline angiographic TIMI flow and TIMI thrombus scores, angiographic characteristics of the culprit lesion, and concomitant treatments (Table 1). OCT data on thrombus score, area, length, and volume, as well as type of thrombus, were also comparable. With regard to the primary PCI, type of intervention, incidence of distal embolization/no‐reflow, and postprocedural angiographic features were not different in the 2 groups.

Repeat coronary angiography and re‐OCT imaging was performed after a median of 6 [6–7] days of treatment in the intervention arm and after 7 [6–7] days of treatment in the control arm (*P*=0.61) (Table 2). Here, TIMI flow score and angiographic features of the culprit lesion were similar in the 2 groups (Table 1). After repeat OCT evaluation, all patients underwent deferred PCI of the culprit lesion, which was performed with stent implantation in 18 (90%) patients of the intervention arm and in 18 (90%) controls, whereas 2 (10%) and 2 (10%) patients, respectively, underwent plain balloon angioplasty. Coronary stenting during deferred PCI was performed based on the operator’s clinical judgment. At the end of the deferred PCI, a TIMI grade 3 flow was obtained in all patients in the 2 arms, with no distal embolization or transient no‐flow.

**Table 2:** Thrombus Data at Baseline (After Primary PCI) and at Repeat OCT (Posttreatment at the Time of Deferred PCI)

	Rivaroxaban arm, n=20	Control arm, n=20	Statistic*	*P* value
Timing of repeat OCT, d (after primary PCI)	6 [6 to 7] d	7 [6 to 7] d	*U*=219	0.61
Baseline OCT (end of primary PCI)				
Plaque features			χ^2^=0.41	0.52
Plaque erosion	9 (45%)	7 (35%)		
Plaque rupture	11 (55%)	13 (65%)		
Thrombus type			χ^2^=0.11	0.74
Red or mixed	7 (35)	8 (40)		
White	13 (65)	12 (60)		
Thrombus score (no. of quadrants)	99 [79 to 172]	113 [86 to 167]	*U*=396	0.70
Thrombus area (mm^2^)	1.01 [0.60 to 2.05]	1.07 [0.44 to 1.77]	*U*=418	0.82
Thrombus length (mm)	11.35 [7.30 to 13.95]	10.70 [8.10 to 16.20]	*U*=413	0.92
Thrombus volume (mm^3^)	12.94 [7.27 to 27.14]	11.45 [3.90 to 25.75]	*U*=433	0.53
Primary end point				
Thrombus score at repeat OCT (no. of quadrants)	39 [27 to 52]	82 [50 to 111]	*U*=307	0.005
Absolute reduction of thrombus score (no. of quadrants)	66 [44 to 131]	44 [10 to 67]	*U*=486	0.040
Relative reduction of thrombus score (%)	61 [50 to 81]	36 [0 to 50]	*U*=524	0.002
Secondary end points				
Thrombus area at repeat OCT (mm^2^)	0.36 [0.20 to 0.79]	0.93 [0.63 to 1.38]	*U*=303	0.004
Absolute reduction of thrombus area (mm^2^)	0.53 [0.21 to 1.16]	0.27 [−0.18 to 0.86]	*U*=473	0.09
Relative reduction of thrombus area (%)	64 [27 to 76]	22 [−32 to 45]	*U*=536	<0.001
Thrombus length at repeat OCT (mm)	4.50 [3.70 to 7.55]	9.20 [5.05 to 11.65]	*U*=342	0.06
Absolute reduction of thrombus length (mm)	4.20 [1.55 to 10.95]	3.1 [0.65 to 4.85]	*U*=465	0.13
Relative reduction of thrombus length (%)	43 [28 to 71]	29 [8 to 44]	*U*=478	0.07
Thrombus volume at repeat OCT (mm^3^)	2.21 [0.98 to 5.13]	8.23 [2.78 to 12.6]	*U*=307	0.005
Absolute reduction of thrombus volume (mm^3^)	8.68 [5.12 to 18.22]	3.46 [0.26 to 16.10]	*U*=482	0.051
Relative reduction of thrombus volume (%)	77 [60 to 91]	39 [0 to 60]	*U*=556	0.001
Thrombus type at repeat OCT			χ^2^=0.14	0.71
Red or mixed	4 (20)	5 (25)		
White	16 (80)	15 (75)		

Data are expressed as number (percentage) or median [interquartile range]. Absolute reduction was calculated by subtracting the follow‐up value from the baseline value. Relative reduction was obtained by dividing the absolute reduction by the baseline value. OCT indicates optical coherence tomography; and PCI, percutaneous coronary intervention.

* The Statistic column reports the value and type of statistical test used for between‐group comparisons: *U*=Mann‐Whitney *U* test statistic.

### Primary End Points

**Figure 4:**
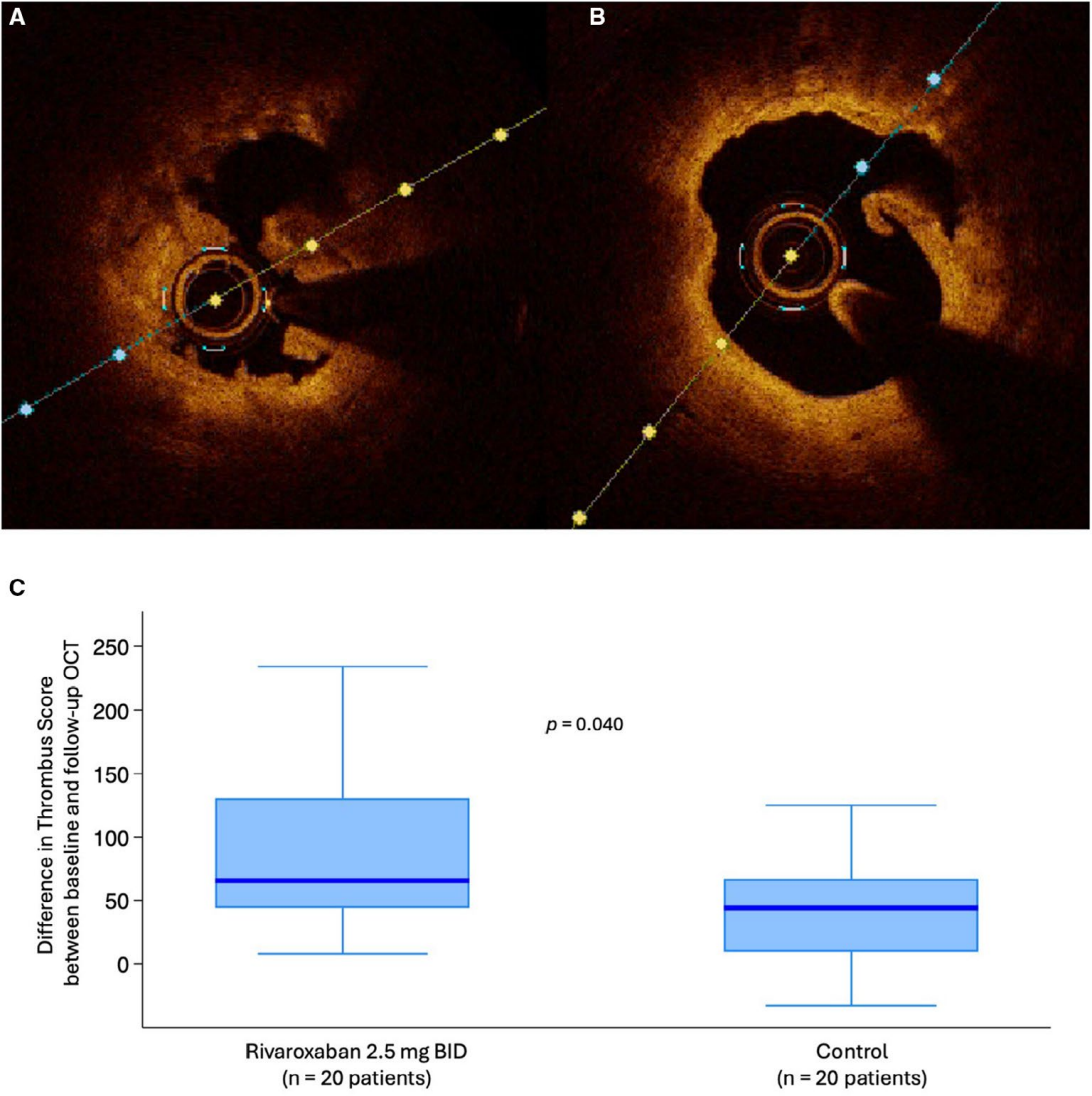
Changes of thrombus score between baseline and follow‐up OCT. **A**, OCT imaging demonstrating a large thrombus burden in the coronary artery at baseline. **B**, OCT imaging of the same coronary section after 6 days of treatment, illustrating thrombus reduction. **C**, Primary study end point with a boxplot comparing the Δ thrombus score between baseline and posttreatment OCT in the 2 arms (rivaroxaban 2.5 mg BID group vs control group). OCT indicates optical coherence tomography.

The thrombus score at re‐OCT imaging, performed after 5 to 7 days of treatment, was significantly lower in the rivaroxaban versus control arm (39 [27–52] versus 82 [50–111] units, *P*=0.005) (Table 2; Figure 4). Posttreatment reductions of the thrombus score compared with baseline were significantly greater with rivaroxaban use (median absolute reduction: 66 [44–131] versus 44 [10–67] units in controls, *P*=0.040; median relative reduction: 61% [50%–81%] versus 36% [0%–50%], *P*=0.002). Thrombus score reduction was consistent among patients presenting with either plaque rupture or erosion, with greater median absolute reductions observed in those treated with rivaroxaban compared with control: 77 [48–95] versus 61 [18–125] units in erosion, and 54 [33–159] versus 37 [1–56] units in rupture, corresponding to median percentage reductions of 63% [57%–67%] versus 52% [14%–65%] and 58% [47%–81%] versus 34% [1.6%–44%], respectively. We performed a sensitivity analysis using permutation‐based tests for the primary end point showing consistent results (Table S1).

### Secondary End Points

**Figure 5:**
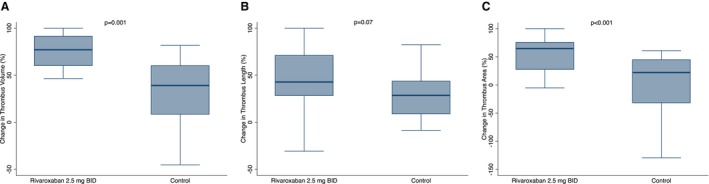
Relative changes of thrombus volume, thrombus area, and thrombus length between baseline and posttreatment optical coherence tomography analyses. **A**, Boxplot comparing the relative reduction of thrombus volume between baseline and posttreatment optical coherence tomography in the 2 arms. **B**, Boxplot comparing the relative reduction of thrombus length between baseline and posttreatment optical coherence tomography in the 2 arms. **C**, Boxplot comparing the relative reduction of thrombus area between baseline and posttreatment optical coherence tomography in the 2 arms.

Patients in the rivaroxaban group showed a significantly lower posttreatment thrombus area (0.36 [0.20–0.79] mm^2^ versus 0.93 [0.63–1.38] mm^2^ in controls, *P*=0.004), numerically higher posttreatment median absolute reduction of thrombus area compared with baseline (0.53 [0.21–1.16] mm^2^ versus 0.27 [−0.18 to 0.86] mm^2^, *P*=0.09), and significantly greater median relative reduction of thrombus area (64% [27%–76%] versus 22% [−32% to 45%], *P*<0.001) (Figure 5; Table 2). Similarly, we observed with rivaroxaban use a numerically lower posttreatment absolute value of thrombus length (4.50 [3.70–7.55] mm versus 9.20 [5.05–11.65] mm, *P*=0.06), and numerically higher reductions of thrombus length compared with baseline (absolute reduction: 4.2 [1.55–10.95] mm versus 3.1 [0.65–4.85] mm, *P*=0.13; relative reduction: 43% [28%–71%] versus 29% [8%–44%], *P*=0.07). Additionally, patients in the rivaroxaban arm posttreatment demonstrated a significantly lower thrombus volume (2.21 [0.98–5.13] mm^3^ versus 8.23 [2.78–12.6] mm^3^, *P*=0.005), a numerically greater absolute decrease of thrombus volume compared with baseline (8.68 [5.12–18.22] mm^3^ versus 3.46 [0.26–16.10] mm^3^, *P*=0.051), and a significantly higher relative reduction of thrombus volume (77% [60%–91%] versus 39% [0%–60%], *P*=0.001). We performed sensitivity analyses using permutation‐based tests also for the secondary OCT end points showing consistent results (Table S1).

Description of thrombus composition at baseline and after treatment is shown in Figure S1. In the re‐OCT assessment, there was in both arms a shift toward a higher proportion of white thrombus and fewer cases of red/mixed thrombus.

With regard to clinical outcome at 30 days (Table 3), 1 patient in the control group suffered from re‐AMI, and 2 patients in the rivaroxaban arm had a nonmajor bleeding event (BARC 2). No patients in the 2 groups died or had a stroke, life‐threatening bleeding, intracranial hemorrhage, or significant hemoglobin drop.

**Table 3:** Clinical Outcomes at 30 Days

	Rivaroxaban arm, n=20	Control arm, n=20	*P* value
MACE	0 (0)	1 (5)	0.34
Death	0	0	…
MI	0	1 (5)	0.34
Stroke or TIA	0	0	…
TVR	0	0	…
Bleeding events			0.15
BARC 1	0	0	
BARC 2	2 (10)	0	
BARC >2	0	0	

Data are expressed as absolute numbers (percentage).

BARC indicates Bleeding Academic Research Consortium; MACE, major adverse cardiovascular events; MI, myocardial infarction; TIA, transient ischemic attack; and TVR, target vessel revascularization.

## DISCUSSION

ARISE‐ARMYDA 7 is a mechanistic, randomized pilot study exploring a new strategy for managing patients with STEMI and LCTB, potentially addressing ongoing challenges such as distal embolization and microvascular obstruction during primary PCI. Despite advances in primary PCI, these complications can limit the success of the infarct‐related artery reperfusion. Our study was focused on whether adding low‐dose rivaroxaban to standard DAPT with aspirin plus ticagrelor, alongside deferred stenting, could enhance thrombus resolution. As compared with patients on DAPT alone, those also receiving rivaroxaban showed greater reductions in thrombus score and thrombus volume. Importantly, this benefit was achieved without increase in major bleeding, reinforcing the potential safety of this strategy in appropriately selected patients.

Managing STEMI in the presence of LCTB requires balancing the need to improve myocardial perfusion while minimizing procedural risks. Thrombus formation is a complex process involving both platelet activation and fibrin generation, with thrombin playing a central role in clot formation. Although DAPT remains the standard of care, the additional use of anticoagulant agents might be beneficial by interfering also on fibrin generation.^8^, ^9^ Historical data from the warfarin era had suggested that adding anticoagulant drugs to conventional antithrombotic treatment after MI reduces the recurrence of thrombotic events. Such an approach significantly increased the bleeding risk, and this has made it unsuitable for a routine use in patients with ACS.^10^ However, the dual function of thrombin, as platelet activator and fibrin generator, suggests that targeting both pathways could be an effective strategy.^8^, ^9^ Thus, the introduction of direct oral anticoagulants, characterized by a higher safety profile, has reopened such an issue when the dual‐pathway inhibition specifically warranted by low‐dose rivaroxaban was evaluated in clinical studies. In particular, rivaroxaban 2.5 or 5 mg BID was tested in the ATLAS ACS 2‐TIMI 51 trial and added to DAPT (aspirin plus clopidogrel) in patients with recent ACS. Its use decreased the occurrence of cardiovascular death, myocardial infarction, and stroke during follow‐up, at the price of increased risk of major nonfatal bleeding.^11^

ARISE‐ARMYDA 7 supports and expands these findings, because it is the first mechanistic study aimed at specifically evaluating and demonstrating a thrombus burden decrease in the infarct‐related artery using an oral anticoagulant drug in the acute phase of STEMI. Notably, a relevant distinction in the study protocol between ARISE‐ARMYDA 7 and ATLAS ACS 2‐TIMI 51 lies in the timing of rivaroxaban initiation (immediately after primary PCI in ARISE‐ARMYDA 7 versus a delayed start, 5 days postprocedure, in ATLAS ACS 2‐TIMI 51). This earlier pharmacological intervention in our study may have enhanced endogenous thrombolysis when the thrombus is less organized, maximizing the antithrombotic effect in the acute phase of STEMI (eg, when the thrombotic risk is high) and overcomes the bleeding risk. A key aspect of any antithrombotic strategy is ensuring the patient’s safety, and in our investigation, the use of rivaroxaban for 6 days was not associated with increased occurrence of major bleeding events, intracranial hemorrhages, or fatalities, suggesting that this approach, given in appropriately selected patients, may be well‐tolerated. The ability of rivaroxaban to act both as anticoagulant drug and antiplatelet agent by reducing thrombin‐mediated platelet activation has been documented in experimental models.^17^ However, with regard to the platelet inhibition by rivaroxaban, observational data suggested a dose‐dependent response, and there are limited mechanistic data on the topic from humans receiving low‐dose rivaroxaban. For these reasons, the exact way in which low‐dose rivaroxaban provides clinical benefits, as observed in the COMPASS (Cardiovascular Outcomes for People Using Anticoagulation Strategies) trial, remains an open issue.^18^

Alternative approaches to LCTB management, such as intracoronary fibrinolysis, have shown promising, but inconclusive results. A meta‐analysis of 8 trials involving 1208 patients demonstrated encouraging findings: intracoronary fibrinolytic therapy in patients with AMI undergoing primary PCI was associated with significant improvement of coronary flow markers and a nonsignificant lower risk of major adverse cardiovascular events.^19^ However, these studies were individually focused on surrogate outcomes derived from invasive hemodynamic/angiographic measures^20^, ^21^, ^22^, ^23^ or from noninvasive imaging techniques,^21^ which indirectly assessed the effects of therapy. Conversely, advanced imaging techniques may play a pivotal role in refining treatment strategies by providing detailed thrombus characterization.^24^ In our study we used OCT, an invasive technique able to provide a precise analysis of thrombus burden. Of note, OCT has shown superior accuracy in quantifying thrombus burden versus conventional angiography.^25^ The use of such a sensitive and specific tool allowed us to quantify posttreatment changes of thrombus score, thrombus volume, area, and length versus baseline, providing a direct causal link between the intervention and its therapeutic effect.

Beyond pharmacological therapy, a mechanical strategy, including thrombus aspiration during primary PCI and deferred re‐PCI, can also play a role in managing patients with STEMI and LCTB, although the effectiveness of these approaches remains debated. The TAPAS (Thrombus Aspiration During Percutaneous Coronary Intervention in Acute Myocardial Infarction), TASTE MI (Thrombus Aspiration in ST‐Elevation Myocardial Infarction), and TOTAL (Trial of Routine Aspiration Thrombectomy With PCI Versus PCI Alone in Patients With STEMI) trials and their meta‐analyses found that a routine thrombus aspiration does not improve long‐term outcomes and increases the risk of stroke.^26^, ^27^, ^28^ However, a selective use of thrombus aspiration in specific situations, such as in patients with significant thrombus burden or no‐reflow, has shown beneficial effects, including better myocardial perfusion when used in a targeted manner. In ARISE‐ARMYDA 7, 65% of patients at the time of primary PCI underwent manual thrombus aspiration. Such strategy successfully restored the TIMI flow, without any major adverse event. This may support the selective use of thrombus aspiration as a complementary tool alongside pharmacological antithrombotic therapy in patients with an LCTB. Stent implantation is another key aspect in managing patients with LCTB. Although direct stenting in patients with AMI undergoing primary PCI is able to directly compress the thrombus, it may also cause complications, such as distal embolization and microvascular obstruction or stent malapposition, all conditions potentially impairing clinical outcome. Deferred stenting offers a safer alternative, because it may allow an antithrombotic therapy‐related thrombus reduction before placing the device. This approach was associated with improvement of coronary flow recovery and left ventricular function, especially in patients with large thrombus burdens.^29^ In ARISE‐ARMYDA 7, deferred stenting was implemented as part of the study protocol and demonstrated clear benefits. The thrombus burden decreased after 5 to 7 days of treatment, with a 36% reduction of thrombus score and a 39% reduction of thrombus volume in the control group receiving aspirin plus ticagrelor and even greater reductions with rivaroxaban addition (61% and 77%, respectively). Importantly, deferred stenting maintained a strong safety profile, with no abrupt vessel closure, acute thrombotic vessel occlusion, distal embolization, or no‐reflow observed in the context of primary PCI or before reintervention. These findings highlight deferred stenting as a safe and effective strategy for managing patients with STEMI with LCTB, especially when combined with an appropriate antithrombotic regimen.

Although ARISE‐ARMYDA 7 offers evidence supporting rivaroxaban‐based regimens and deferred stenting, its limitations must be acknowledged. First, the study has a limited sample size. Moreover, it has a single‐center design and included patients at non‐high bleeding risk, both aspects potentially affecting the generalizability of the findings. Additionally, the open‐label design could introduce a bias, although the assessment of OCT‐related end points by blinded investigators in an external and independent core laboratory surely mitigates this concern. The lack of prolonged rivaroxaban administration and long‐term follow‐up limit the ability to assess the impact of this drug in terms of thrombotic events avoided and bleeding complications caused. Thus, future larger studies are needed to investigate whether and how the thrombus reduction by rivaroxaban given after primary PCI in preparation to deferred coronary stenting translates into lower adverse clinical events, mainly in terms of lower procedural ischemic complications during reintervention. Notably, investigating the interplay between thrombus composition and therapeutic response could further refine patient selection and optimize outcomes. Finally, our study was not intended to perform a comparison between immediate versus deferred PCI, because it was designed as a mechanistic pilot trial exclusively focused on thrombus resolution through intensification of the antithrombotic approach.

In conclusion, the ARISE‐ARMYDA 7 trial provides new evidence on an early use of low‐dose rivaroxaban in patients with STEMI with LCTB undergoing primary PCI and deferred stenting. By demonstrating a superior thrombus resolution, our study highlights the potential of a dual‐pathway inhibition approach on top of standard DAPT to potentially improve outcome in a challenging patients population, representing a significant step forward in managing complex STEMI cases.

## Sources of Funding

None.

## Disclosures

G.P.: speaker/consultant fees from Amgen, Sanofi, Novartis, Daichi Sankyo, Amarin, Aurora BioPharma, Malesci, PIAM, Boheringer Ingheleim, Bayer, Pfizer/BMS, Astra Zeneca, Biotronik, Terumo, Medtronic, Abbott, Edwards, Amicus, Novo Nordisk, Chiesi. R.V.: consultant for Abbott Vascular and Medtronic; received lecturing fees from Abbott Vascular, Abiomed, Daiichi Sankyo, Edwards Lifesciences, Medtronic, Novartis, and Philips; and served as a member of advisory boards for Amarin and Amgen. M.G.M.: speaker/consultant fees from Amgen, PIAM, Boheringer Ingheleim, Pfizer/BMS, Astra Zeneca, and Edwards Lifesciences. L.G., G.A., M.S., V.G., M.V., M.B., E.I., R.Rosso, F.D.C., D.D.A., R.Rippa: no disclosures.

## Supporting information

Figure S1

## References

[1] Libby P . Mechanisms of acute coronary syndromes and their implications for therapy. N Engl J Med. 2013;368:2004–2013. doi: 10.1056/NEJMra1216063 23697515

[2] Ibanez B , James S , Agewall S , Antunes MJ , Bucciarelli‐Ducci C , Bueno H , Caforio ALP , Crea F , Goudevenos JA , Halvorsen S , et al. 2017 ESC guidelines for the management of acute myocardial infarction in patients presenting with ST‐segment elevation: the task force for the management of acute myocardial infarction in patients presenting with ST‐segment elevation of the European Society of Cardiology (ESC). Eur Heart J. 2018;39:119–177. doi: 10.1093/eurheartj/ehx393 28886621

[3] Byrne RA , Rossello X , Coughlan JJ , Barbato E , Berry C , Chieffo A , Claeys MJ , Dan G‐A , Dweck MR , Galbraith M , et al. 2023 ESC guidelines for the management of acute coronary syndromes: developed by the task force on the management of acute coronary syndromes of the European Society of Cardiology (ESC). Eur Heart J. 2023;44:3720–3826. doi: 10.1093/eurheartj/ehad191 37622654

[4] Fokkema ML , Vlaar PJ , Svilaas T , Vogelzang M , Amo D , Diercks GFH , Suurmeijer AJH , Zijlstra F . Incidence and clinical consequences of distal embolization on the coronary angiogram after percutaneous coronary intervention for ST‐elevation myocardial infarction. Eur Heart J. 2009;30:908–915. doi: 10.1093/eurheartj/ehp033 19224928

[5] Lønborg J , Kelbæk H , Helqvist S , Holmvang L , Jørgensen E , Saunamäki K , Kløvgaard L , Kaltoft A , Bøtker HE , Lassen JF , et al. The impact of distal embolization and distal protection on long‐term outcome in patients with ST elevation myocardial infarction randomized to primary percutaneous coronary intervention – results from a randomized study. Eur Heart J Acute Cardiovasc Care. 2015;4:180–188. doi: 10.1177/2048872614543780 25013089

[6] Isaaz K , Robin C , Cerisier A , Lamaud M , Richard L , Da Costa A , Sabry MH , Gerenton C , Blanc JL . A new approach of primary angioplasty for ST‐elevation acute myocardial infarction based on minimalist immediate mechanical intervention. Coron Artery Dis. 2006;17:261–269. doi: 10.1097/00019501-200605000-00010 16728877

[7] Qiao J , Pan L , Zhang B , Wang J , Zhao Y , Yang R , Du H , Jiang J , Jin C , Xiong E . Deferred versus immediate stenting in patients with ST‐segment elevation myocardial infarction: a systematic review and meta‐analysis. J Am Heart Assoc. 2017;6:e004838. doi: 10.1161/JAHA.116.004838 28275065 PMC5524015

[8] De Caterina R , Husted S , Wallentin L , Andreotti F , Arnesen H , Bachmann F , Baigent C , Huber K , Jespersen J , Kristensen SD , et al. European Society of Cardiology Working Group on thrombosis task force on anticoagulants in heart disease. General mechanisms of coagulation and targets of anticoagulants (section I). Position paper of the ESC working group on thrombosis‐‐task force on anticoagulants in heart disease. Thromb Haemost. 2013;109:569–579. doi: 10.1160/TH12-10-0772 23447024

[9] Tamura N , Kitajima I , Kawamura Y , Toda E , Eguchi Y , Ishida H , Goto S . Important regulatory role of activated platelet‐derived procoagulant activity in the propagation of thrombi formed under arterial blood flow conditions. Circ J. 2009;73:540–548. doi: 10.1253/circj.CJ-08-0465 19179771

[10] Andreotti F , Testa L , Biondi‐Zoccai GGL , Crea F . Aspirin plus warfarin compared to aspirin alone after acute coronary syndromes: an updated and comprehensive meta‐analysis of 25,307 patients. Eur Heart J. 2006;27:519–526. doi: 10.1093/eurheartj/ehi485 16143706

[11] Mega JL , Braunwald E , Wiviott SD , Bassand J‐P , Bhatt DL , Bode C , Burton P , Cohen M , Cook‐Bruns N , Fox KAA , et al. Rivaroxaban in patients with a recent acute coronary syndrome. N Engl J Med. 2012;366:9–19. doi: 10.1056/NEJMoa1112277 22077192

[12] Gibson CM , de Lemos JA , Murphy SA , Marble SJ , McCabe CH , Cannon CP , Antman EM , Braunwald E ; TIMI Study Group . Combination therapy with abciximab reduces angiographically evident thrombus in acute myocardial infarction: a TIMI 14 substudy. Circulation. 2001;103:2550–2554. doi: 10.1161/01.cir.103.21.2550 11382722

[13] Prati F , Guagliumi G , Mintz GS , Costa M , Regar E , Akasaka T , Barlis P , Tearney GJ , Jang I‐K , Arbustini E , et al. Expert review document part 2: methodology, terminology and clinical applications of optical coherence tomography for the assessment of interventional procedures. Eur Heart J. 2012;33:2513–2520. doi: 10.1093/eurheartj/ehs095 22653335 PMC3470836

[14] Prati F , Capodanno D , Pawlowski T , Ramazzotti V , Albertucci M , La Manna A , Di Salvo M , Gil RJ , Tamburino C . Local delivery versus intracoronary infusion of abciximab in patients with acute coronary syndromes. JACC Cardiovasc Interv. 2010;3:928–934. doi: 10.1016/j.jcin.2010.05.017 20850091

[15] Mehran R , Rao SV , Bhatt DL , Gibson CM , Caixeta A , Eikelboom J , Kaul S , Wiviott SD , Menon V , Nikolsky E , et al. Standardized bleeding definitions for cardiovascular clinical trials. Circulation. 2011;123:2736–2747. doi: 10.1161/CIRCULATIONAHA.110.009449 21670242

[16] Jia H , Dai J , Hou J , Xing L , Ma L , Liu H , Xu M , Yao Y , Hu S , Yamamoto E , et al. Effective anti‐thrombotic therapy without stenting: intravascular optical coherence tomography‐based management in plaque erosion (the EROSION study). Eur Heart J. 2017;38:792–800. doi: 10.1093/eurheartj/ehw381 27578806

[17] Petzold T , Thienel M , Dannenberg L , Mourikis P , Helten C , Ayhan A , M’Pembele R , Achilles A , Trojovky K , Konsek D , et al. Rivaroxaban reduces arterial thrombosis by inhibition of FXa‐driven platelet activation via protease activated Receptor‐1. Circ Res. 2020;126:486–500. doi: 10.1161/CIRCRESAHA.119.315099 31859592

[18] Eikelboom JW , Connolly SJ , Bosch J , Dagenais GR , Hart RG , Shestakovska O , Diaz R , Alings M , Lonn EM , Anand SS , et al. Rivaroxaban with or without aspirin in stable cardiovascular disease. N Engl J Med. 2017;377:1319–1330. doi: 10.1056/NEJMoa1709118 28844192

[19] Sahami N , Akl E , Sanjanwala R , Shah AH . Safety and efficacy of low‐dose intracoronary thrombolysis during primary percutaneous coronary intervention in patients with ST elevation myocardial infarction: a meta‐analysis of randomized trials. Curr Probl Cardiol. 2024;49:102616. doi: 10.1016/j.cpcardiol.2024.102616 38718936

[20] Sezer M , Oflaz H , Gören T , Okçular İ , Umman B , Nişanci Y , Bilge AK , Şanli Y , Meriç M , Umman S . Intracoronary streptokinase after primary percutaneous coronary intervention. N Engl J Med. 2007;356:1823–1834. doi: 10.1056/NEJMoa054374 17476008

[21] Sezer M , Cimen A , Aslanger E , Elitok A , Umman B , Buğra Z , Yormaz E , Türkmen C , Adalet IS , Nişanci Y , et al. Effect of intracoronary streptokinase administered immediately after primary percutaneous coronary intervention on long‐term left ventricular infarct size, volumes, and function. J Am Coll Cardiol. 2009;54:1065–1071. doi: 10.1016/j.jacc.2009.04.083 19744615

[22] Greco C , Pelliccia F , Tanzilli G , Tinti MD , Salenzi P , Cicerchia C , Schiariti M , Franzoni F , Speziale G , Gallo P , et al. Usefulness of local delivery of thrombolytics before thrombectomy in patients with ST‐segment elevation myocardial infarction undergoing primary percutaneous coronary intervention (the delivery of thrombolytics before thrombectomy in patients with ST‐segment elevation myocardial infarction undergoing primary percutaneous coronary intervention [DISSOLUTION] randomized trial). Am J Cardiol. 2013;112:630–635. doi: 10.1016/j.amjcard.2013.04.036 23711809

[23] Gibson CM , Kumar V , Gopalakrishnan L , Singh P , Guo J , Kazziha S , Devireddy C , Pinto D , Marshall JJ , Stouffer GA , et al. Feasibility and safety of low‐dose intra‐coronary Tenecteplase during primary percutaneous coronary intervention for ST‐elevation myocardial infarction (ICE T‐TIMI 49). Am J Cardiol. 2020;125:485–490. doi: 10.1016/j.amjcard.2019.11.018 31870492

[24] Amabile N , Hammas S , Fradi S , Souteyrand G , Veugeois A , Belle L , Motreff P , Caussin C . Intra‐coronary thrombus evolution during acute coronary syndrome: regression assessment by serial optical coherence tomography analyses. Eur Heart J Cardiovasc Imaging. 2015;16:433–440. doi: 10.1093/ehjci/jeu228 25428947

[25] Porto I , Mattesini A , Valente S , Prati F , Crea F , Bolognese L . Optical coherence tomography assessment and quantification of intracoronary thrombus: status and perspectives. Cardiovasc Revasc Med. 2015;16:172–178. doi: 10.1016/j.carrev.2015.01.007 25681257

[26] Svilaas T , Vlaar PJ , van der Horst IC , Diercks GFH , de Smet BJGL , van den Heuvel AFM , Anthonio RL , Jessurun GA , Tan E‐S , Suurmeijer AJH , et al. Thrombus aspiration during primary percutaneous coronary intervention. N Engl J Med. 2008;358:557–567. doi: 10.1056/NEJMoa0706416 18256391

[27] Fröbert O , Lagerqvist B , Gudnason T , Thuesen L , Svensson R , Olivecrona GK , James SK . Thrombus aspiration in ST‐elevation myocardial infarction in Scandinavia (TASTE trial). A multicenter, prospective, randomized, controlled clinical registry trial based on the Swedish angiography and angioplasty registry (SCAAR) platform. Study design and rationale. Am Heart J. 2010;160:1042–1048. doi: 10.1016/j.ahj.2010.08.040 21146656

[28] Jolly SS , Cairns JA , Lavi S , Cantor WJ , Bernat I , Cheema AN , Moreno R , Kedev S , Stankovic G , Rao SV , et al. Thrombus aspiration in patients with high thrombus burden in the TOTAL trial. J Am Coll Cardiol. 2018;72:1589–1596. doi: 10.1016/j.jacc.2018.07.047 30261959

[29] De Maria GL , Alkhalil M , Oikonomou EK , Wolfrum M , Choudhury RP , Banning AP . Role of deferred stenting in patients with ST elevation myocardial infarction treated with primary percutaneous coronary intervention: a systematic review and meta‐analysis. J Interv Cardiol. 2017;30:264–273. doi: 10.1111/joic.12380 28370496

