# Supplementary material for: Reduction of Thrombus Burden With Short‐Term, Low‐Dose Rivaroxaban Use in Acute Myocardial Infarction: The ARISE‐ARMYDA 7 Randomized Trial
Source: J Am Heart Assoc. 2025 Nov 6;14(22):e041993. doi: 10.1161/JAHA.125.041993 (PMC12887225; doi:10.1161/JAHA.125.041993)
Supplement: Supplementary file 1 — Figure S1 [file JAH3-14-e041993-s001.zip › jah311445-sup-0001-Supplement.pdf]

SUPPLEMENTAL MATERIAL

Table S1. Results of Permutation-Based Sensitivity Analyses for Primary and Secondary OCT endpoints.

	**Permutation Test p value***	**Monte Carlo SE †**	**95% CI p value †**	**Cliff's Delta ‡**
Primary endpoint				
Thrombus Score at repeat OCT (n. of quadrants)	0.004	0.001	0.002–0.005	0.38
Absolute reduction of Thrombus Score (n. of quadrants)	0.035	0.002	0.032–0.039	0.51
Relative reduction of Thrombus Score (%)	0.001	<0.001	<0.001–0.002	0.57
Secondary endpoints				
Thrombus Area at repeat OCT (mm2)	0.003	0.001	0.002–0.005	0.53
Absolute reduction of Thrombus Area (mm2)	0.09	0.003	0.092–0.103	0.31
Relative reduction of Thrombus Area (%)	0.001	<0.001	0.001–0.002	0.63
Thrombus Length at repeat OCT (mm)	0.07	0.003	0.067–0.077	0.34
Absolute reduction of Thrombus Length (mm)	0.14	0.003	0.130–0.143	0.28
Relative reduction of Thrombus Length (%)	0.07	0.003	0.061–0.070	0.34
Thrombus Volume at repeat OCT (mm3)	0.004	0.001	0.003–0.005	0.51
Absolute reduction of Thrombus Volume (mm3)	0.055	0.002	0.051–0.059	0.36
Relative reduction of Thrombus Volume (%)	<0.001	<0.001	<0.000–0.001	0.73

*The two-sided p-values shown in the table were calculated using a Monte Carlo permutation test with 10,000 iterations and reflect the probability that the observed differences occurred by chance. †The Monte Carlo standard error (SE) quantifies the uncertainty of the estimated p-value due to the random nature of the permutations, while the 95% confidence interval (CI) provides the range within which the true p-value is expected to fall with 95% confidence. ‡Cliff's delta is a non-parametric measure of effect size that ranges from –1 to +1 and indicates how much the values in one group tend to be higher or lower than those in the other. A value of 0 suggests no difference, while values further from zero indicate larger effects. Conventionally, absolute values of 0.147, 0.33, and 0.474 are considered thresholds for negligible, small, moderate, and large effects, respectively.

OCT= Optical Coherence Tomography.

Figure S1. Evolution of thrombus type before and after treatment.

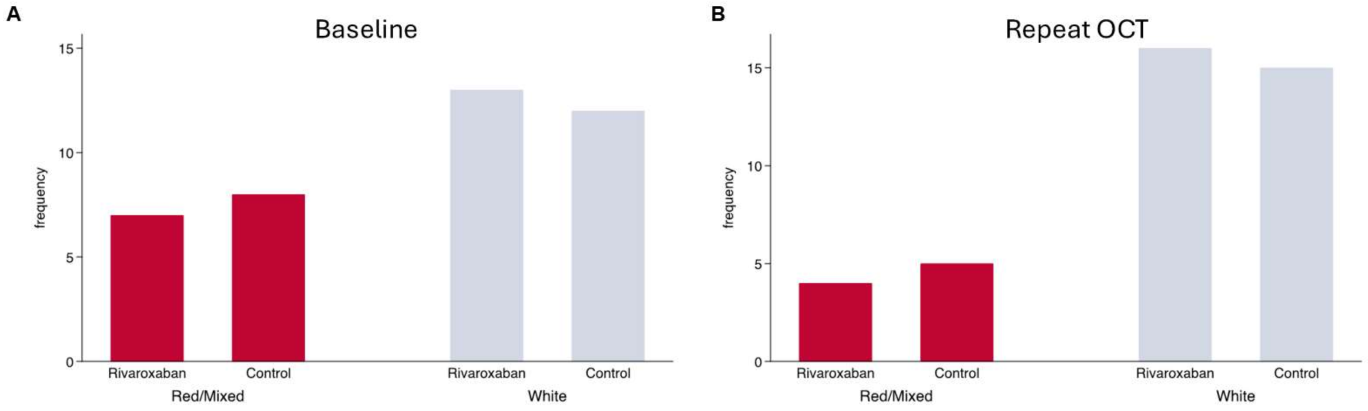

OCT imaging of the thrombus type at baseline at the time of primary PCI (**A**) and after 5 to 7 days of treatment (**B**).

OCT= Optical Coherence Tomography; PCI = Percutaneous Coronary Intervention
